# Development and evaluation of clinically effective “*APOE* Genotyping SNP Array System” for ARIA risk assessment in Alzheimer's Disease patients

**DOI:** 10.1002/alz70856_102590

**Published:** 2025-12-24

**Authors:** Kazuya Kurachi, Nozomu Watanabe, Osamu Shibata, Shoji Okajima, Jeffrey Albrecht, Masahiro Ieko

**Affiliations:** ^1^ LabCorp Japan, G.K., Chuo‐ku, Tokyo, Japan; ^2^ Consumer Genetics, Laboratory Corporation of America Holdings, Chicago, IL, USA; ^3^ Sapporo University of Health Sciences, Sapporo, Hokkaido, Japan

## Abstract

**Background:**

The anti‐amyloid‐β (Aβ) antibody drugs for Alzheimer's Disease (AD), “Lecanemab” and “Donanemab,” have been approved in the United States, Japan, and other countries, and their administration has commenced in clinical treatments. One of the common side effects, Amyloid‐Related Imaging Abnormalities (ARIA), have been reported for these anti‐Aβ antibody drugs, with the incidence of ARIA varying based on apolipoprotein E (*APOE*) genotype. In both the United States and Japan, it is recommended that *APOE* genotyping tests be conducted to identify the risk of ARIA prior to these AD drug treatments. Laboratory Corporation of America Holdings offers a quality‐controlled *APOE* Genotyping Single Nucleotide Polymorphism (SNP) Array, “*APOE* Alzheimer's Disease Risk,” as a Laboratory Developed Test (LDT). In this study, we confirmed that the test is useful for assessing the risk of ARIA in patients undergoing treatment with anti‐Aβ antibody drugs.

**Method:**

DNA samples derived from whole blood specimens are used. Custom genotyping arrays are employed for two common variants in the *APOE* gene: rs429358 and rs7412. Three prevalent combinations of these genetic variants include ε2 (Cys130, Cys176), ε3 (Cys130, Arg176), and ε4 (Arg130, Arg176). Individuals are classified into one of the following genotypes: ε2/ε2, ε2/ε3, ε2/ε4, ε3/ε3, ε3/ε4, or ε4/ε4.

**Result:**

We confirmed the concordance of *APOE* genotypes between the *APOE* Genotyping SNP Array and the Polymerase Chain Reaction (PCR) method. The PCR method was performed in the same laboratory with the same reagents, equipment, and procedures as those employed in the Donanemab Phase III clinical trial (TRAILBLAZER‐ALZ 2). A total of 54 samples were tested, and the concordance rate between the *APOE* Genotyping SNP Array and the PCR method for all six genotypes was 100%, with at least six samples per genotype tested as of January 2025.

**Conclusion:**

It was demonstrated that the *APOE* Genotyping SNP Array is a valuable tool for assessing the risk of ARIA in patients receiving anti‐Aβ antibody drugs. In the future, it is anticipated that the test will be widely utilized to evaluate the risk of ARIA prior to the administration of anti‐Aβ antibody drugs.

